# Quality Control of Glehniae Radix, the Root of *Glehnia Littoralis* Fr. Schmidt ex Miq., Along its Value Chains

**DOI:** 10.3389/fphar.2021.729554

**Published:** 2021-10-04

**Authors:** Yuan Chen, Lujing Lei, Yaqiong Bi, Linlin Jiang, Wenfang Guo, Jianhua Wang, Minhui Li

**Affiliations:** ^1^ Department of Pharmacy, Inner Mongolia Medical University, Hohhot, China; ^2^ Inner Mongolia Institute of Traditional Chinese Medicine, Hohhot, China; ^3^ Inner Mongolia Key Laboratory of Characteristic Geoherbs Resources Protection and Utilization, Baotou, China; ^4^ Baotou Medical College, Baotou, China

**Keywords:** glehniae radix, value chain, quality, stakeholder, traditional Chinese medicine

## Abstract

Glehniae Radix (GR) is one of the major medicinal materials in China. The global demand for GR, especially in Asian countries, is constantly increasing, and the supply of wild medicinal materials falls short of the demand. Previous studies have shown that the production and processing modes of different value chains (VCs) impact the quality of medicinal materials. After 4 years of field and market research, this study includes interviews with stakeholders in the VCs, integrates different types of VCs, and further analyzes the VCs. GR characteristics were also assessed; the length and upper-middle diameter of the collected samples were measured, and the effective components of the samples were determined to rank the GR samples according to their quality. The effective components were further analyzed by the *K*-means clustering method. Concomitantly, the local price (the sales price of the place where the medicinal materials are produced) and market price (the sale price of medicinal materials in the market) of GR in Chifeng, Inner Mongolia, and Anguo, Hebei, were documented, and the ARIMA (Autoregressive Integrated Moving Average) method was used to predict the GR price. Ten VCs are summarized in this article. The results showed that the income of the staff at the beginning of the VC is inadequate. Regarding GR origin, Inner Mongolia GR showed higher quality than that of other areas. As a result, the price of medicinal materials is relatively high, which corresponds to the market price of the survey. The forecast results showed that the market price of GR would increase slightly in the future, which could provide reference for the selection of medicinal materials cultivation in the future. Through the study, it was found that the vertical integration in the VCs of GR could guarantee not only the benefit of the growers but also the traceability of the medicinal materials, which further guarantees the quality of the medicinal materials. However, the complex relationship between the cultivation area and the quality of the medicinal materials is not clear, which should be addressed in future research.

## Introduction

Traditional Chinese medicine (TCM) has been used for thousands of years and continues to play an important role in human health, as it is used by over 80% of the global population (Thomford et al., 2018). The 2020 COVID-19 pandemic revealed differences in the level of healthcare among countries. China adheres to the concept of integrating the theories of traditional Chinese and Western medicine, coordinating their resources. The country has achieved good results in the joint prevention, control, and treatment of COVID-19. This fact, once again, has increased global awareness of the advantages of Chinese medicine in the medical and health industries. The global trade of medicinal plants and their derivatives was estimated at 33 billion USD in 2014, and the World Health Organization (WHO) has estimated that this will increase to 50 trillion USD by 2050. The export of medicinal plants in China increased eight-fold from 2010 to 2015 (Pyakurel et al., 2018; Volenzo and Odiyo, 2020). Traditional Chinese medicines not only occupy an important position in the global medical field but are also used in dietary supplements, food, and other products (Yao et al., 2018; Zhang et al., 2021).

With the gradual increase in market demand for TCM, the production scale is growing, the supply system is gradually diversifying, and supervision and import entry are becoming rigorous in many countries and regions (Pan et al., 2013; Melo et al., 2014). For example, Europe has played a leading role in requiring high-quality herbal (Qu et al., 2018). Quality control and safety evaluation standards in public and private sectors define the admittance criterion before a product is released to the market (Booker et al., 2016a). However, the improvement of standards is beneficial for safety and efficacy and is deemed to add value to a product (Booker et al., 2015).

In order to improve the quality standards of TCM, it is necessary to develop a quality evaluation system for TCM. Traditional methods to identify the quality of medicinal materials mainly rely on their appearance (Gao et al., 2021). With the development of modern analytical techniques (Li and Weng, 2017), a variety of advanced interdisciplinary techniques, including omics, chemical informatics, and network pharmacology, are used to evaluate the quality of medicinal materials (Li et al., 2021). This includes evaluating the quality of medicinal materials by comparing different analytical methods (Booker et al., 2014; Booker et al., 2016b). It should be noted that a comprehensive evaluation should be meticulous when evaluating the quality of medicinal materials.

Value chain (VC) analysis can be used for the quality evaluation of TCM and as a tool to study the status of the TCM industry in the context of global trade (Booker et al., 2012). From a management perspective, VCs can be used to evaluate the correctness and effectiveness of regulations and decisions. In the TCM industry, VCs can be integrated and optimized to improve the quality of the end product. Stakeholders participating in VCs can improve economic performance by the analysis of VCs (Yao et al., 2018). Especially for primary producers, VC correlation analyses are critical (Heinrich, 2015).

A VC describes the transactions and processing of a product from raw materials to the end product, considering manufacturing, distribution, marketing, relationships among various participants involved in the chain, and the share of value added (Booker et al., 2015; Ingram et al., 2018). In this context, the study of VCs focuses on the relationships among participants in the chain (Heinrich, 2015). In recent years, an increasing number of studies have used correlation analyses to integrate the concept of VCs into the production of TCM materials (Booker et al., 2016a; Giacomelli et al., 2017). This type of analysis is useful because, according to different production and circulation modes, investment risk differs among medicinal materials.

**FIGURE 1:**
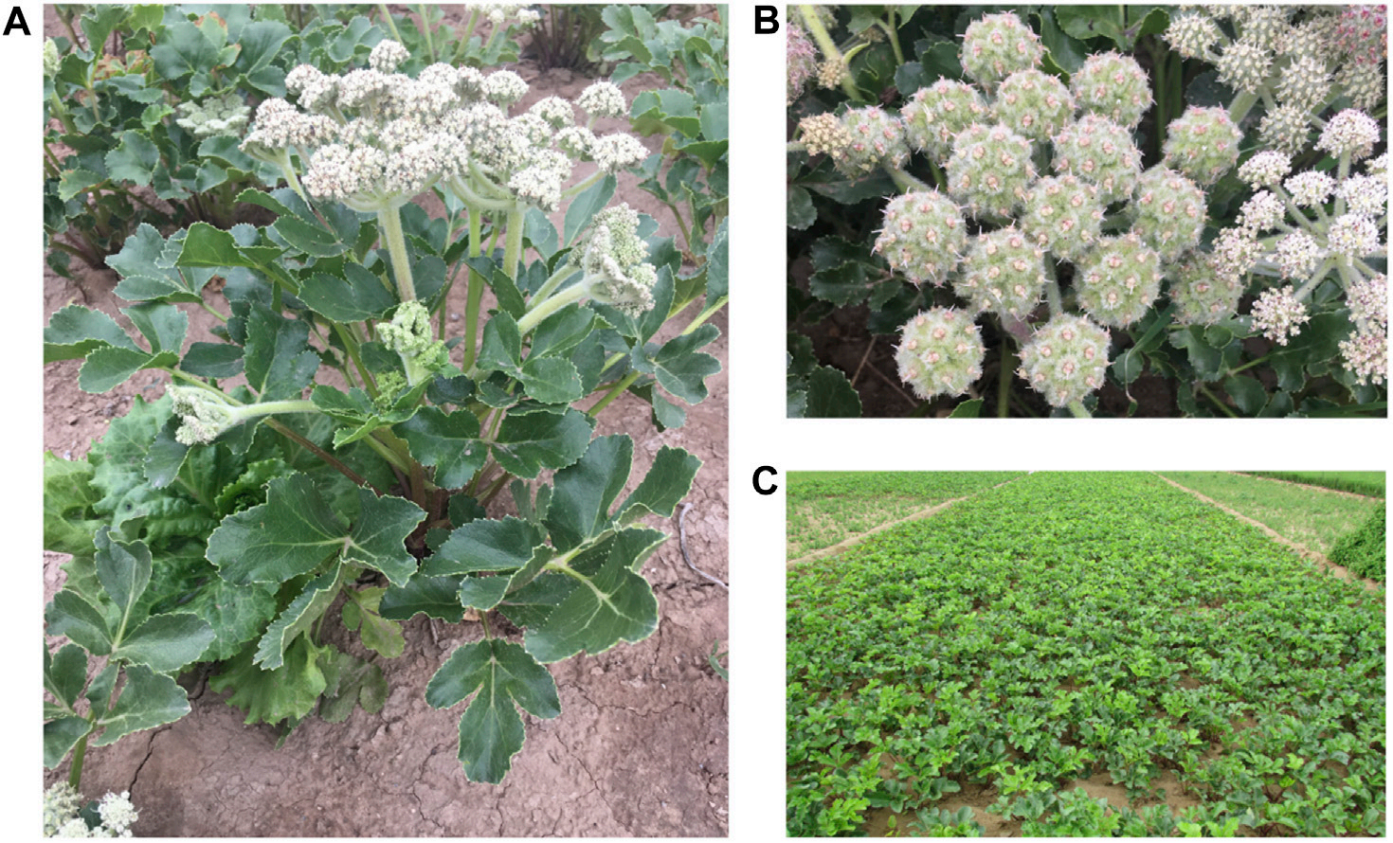
The cultivated of *Glehnia littoralis* Fr. Schmidt ex Miq. **(A)**, umbel details of *Glehnia littoralis* Fr. Schmidt ex Miq. **(B)** and planting base of *Glehnia littoralis* Fr. Schmidt ex Miq. **(C)**.

Glehniae Radix (GR) is the dried root of *Glehnia littoralis* Fr. Schmidt ex Miq., belonging to the family Umbelliferae, as shown in Figure 1. GR has been a popular TCM in China, Japan, and Korea for thousands of years. GR is called Beishashen in China and is used in Chinese and Mongolian medicine to relieve coughs, moisten lungs, and clear lung-heat and as an antiphlogistic for the treatment of respiratory and digestive diseases (Yuan et al., 2002). The active constituents isolated from GR, e.g., coumarins, polysaccharides, lignans, and flavonoids, show antioxidant, anti-inflammatory, antitumor, and immunoregulatory activities (Li et al., 2018). Among these compounds, polysaccharides and coumarins are important active components of GR; they are closely related to the traditional curative effect and modern pharmacological effect of GR (Zhang, 2002; Liu et al., 2013). The main pharmacological effects of GR polysaccharides are immune regulation, anti-tumorigenesis, anti-oxidation, anti-aging, among others (Wang and Su, 2020). Previous studies indicated that the content of polysaccharides could be used as a reference index for evaluating the quality of GR (Zhang, 2002). In addition, Xanthotoxin was list as carcinogen by International Agency for Research on Cancer of World Health Organization in 2017. But there are only animal toxicity related studies (Liu et al., 2004), lack of clinical studies, and lack of limit standards. In order to comprehensively evaluate the medicinal materials with multiple indexes, these two components were selected as the quality index for analyzing the quality of GR in this study. GR is also utilized in cooking. It is a tonic ingredient commonly used in soup-making in southern China, including in Guangdong province. Moreover, it has been found that GR can be used as raw material for ceramic products resistant to Aspergillus flavus (Cheng and Zhou, 2017). GR has a cultivation history of several hundred years in China. According to ancient Chinese bencao, the daodi production area (which means “be out and out”) for GR is Laiyang in Shandong province. Nevertheless, owing to market competition and changes in the supply chain, Chifeng in Inner Mongolia is now the main production area, accounting for 80% of the total GR production, and Chifeng GR is recognized as the highest-quality GR in the market.

In this study, we evaluated: the VCs of GR in Chifeng, Inner Mongolia, along the stages of production (from cultivation and processing) to transfer to the end consumer; the distribution of benefits; the characteristics of different VCs as well as the livelihoods and relationships among various stakeholders in the chains; and the linkage between GR quality and the management of VCs. The results of these analyses are expected to provide a valuable reference for improving the related interests of stakeholders along with VCs and ensuring GR quality. The main research objectives were: 1) to evaluate the GR quality risk in Chifeng, Inner Mongolia, and compare GR quality between Chifeng, Inner Mongolia, Laiyang, Shandong Province and Anguo, Hebei Province; 2) to analyze the venture capital, stakeholders’ interest relationships, and financial performance of GR and to propose strategies to improve quality and safety by integrating and optimizing GR venture capital.

## Materials and Methods

### Fieldwork and Market Survey

Five farmers, seven processors, six owners of TCM distribution stations, two logistics workers, and two business managers of a local medicine enterprise (Rongxingtang Pharmaceutical Co., Ltd). participated in interviews. The fieldwork was performed in 2017–2020, during the GR harvesting period (i.e., from September to November). The survey covered three locations, Chifeng of Inner Mongolia, Laiyang of Shandong Province, and Anguo of Hebei Province, which are the main GR production areas in China. The sites were mainly farmland, TCM trading centers, and TCM distribution stations. The TCM distribution stations were privately owned. The owners were brokers or middlemen, and some also cultivated GR.

A market survey was conducted in November 2020 in Anguo (Heibei Province) and Bozhou City (Anhui Province), the two largest medicinal material distribution centers in China. The main respondents were wholesalers (n = 5) or retailers (n = 5) of GRs. Telephone interviews were also adopted for one wholesaler from Bozhou and one from Anguo, one buyer for a processing plant for herbal pieces (Anhui People’s Traditional Chinese Co., Ltd), and one middleman from a pharmaceutical company. In addition, foreign importers were investigated in March 2021 in the form of a questionnaire to obtain a preliminary understanding of the import of GRs from Japan and South Korea.

Respondents were involved in different stages in the GR VC and were selected randomly. The interviews lasted from 20 min to 1 h in a semi-structured manner, and interviewees were encouraged to provide any relevant information. Interviews were recorded with the consent of the interviewees. The information acquired was cross-validated by the mutual verification (Coslovsky, 2014) of different interviewees, website information, government data, and news reports.

### Plant Material

In total, 48 GR samples were collected during fieldwork and market surveys. The samples were mainly obtained from local farmers or GR retailers or were purchased from medicinal markets. The exported GRs were provided by Rongxingtang Pharmaceutical Co., Ltd. Length, upper middle diameter, moisture, total Ash, acid-insoluble ash, extractum, polysaccharide content, XAT content, five heavy metals and twelve pesticide residues were detected.

### Sample Quality Evaluation

#### Chemical Reagents

Dextrose anhydrate was purchased from the National Institutes for Food and Drug Control (Beijing, China). Acetonitrile was purchased from Beijing Merida Technology Co., Ltd. (Beijing, China). The Im, Ge, Bi, Au, Pb, Cd, As, Hg, and Cu reference materials were purchased from Non-ferrous Metal and Electronic Materials Analysis and Testing Center (Beijing, China). Citrus leaves were purchased from IGGE (Beijing, China). Nitric acid was purchased from Merck (Darmstadt, Germany). The trifluralin, dichlorvos, chlorpyrifos, quintozene, *α*-BHC, *β*-BHC, *γ*-BHC, *δ*-BHC, *pp*'-DDE, *pp*'-DDD, *op*'-DDT, and *pp*'-DDT reference materials were purchased from the Environmental Protection Scientific Research and Monitoring Institute of the Ministry of Agriculture (Being, China). The magnesium sulfate anhydrous >99%, sodium acetate anhydrous >99% were purchased from Aladdin. The acetone was chromatographically pure grade and was purchased from Mreda (Beijing, China). Purified water was obtained using Water Purification Systems (Shenyang, China), all other reagents were of analytical grade.

#### Determination of GR Polysaccharide Content

The water extraction and alcohol-precipitation (80% ethyl alcohol as precipitant) methods were used for sample processing. The GR polysaccharide content of the aqueous solution was determined on a UV-1800 spectrophotometer (Shimadzu, Kyoto, Japan) using the anthrone-sulfuric method. The determination wavelength was 627 nm.

#### Determination of GR XAT Content

Water extraction and alcohol-precipitation (80% ethyl alcohol as precipitant) methods were used for sample processing. The samples were determined by high-performance liquid chromatography (Shimadzu) with octadecyl bonded silica gel as filler. Acetonitrile was used as mobile phase A, and 0.1% phosphoric acid solution as mobile phase B. The detection wavelength was 250 nm.

#### Determination of Extract Content in GR

The GR water-soluble extracts were prepared employing the method of Pharmacopoeia of the People’s Republic of China (ChP, 2020). The GR powders were obtained using heating reflux for 1 h, the extracting solution was dried for 3 h at 105°C, and the weight of residue was used to calculate the extracted content.

#### Determination of Heavy Metals

Pb, Cd, As, Hg, and Cu were determined as per the method of ChP 2020. The GR powders were treated with an ETHOS UP microwave digestion system (Milestone Srl, Sorisole, Italy). The sample solutions were analyzed on an ICAP-RQ ICP-MS (Thermo Fisher Scientific, Waltham, MA, United States ).

#### Determination of Pesticide Residue

The determination of pesticide residue was performed employing the method called QuEnChERS (ChP, 2020). The GR powder was treated with dispersing solid extraction purification pipe (Angela). Chromatographic separation was conducted using a capillary column of 3,000 × 0.25 mm, 0.25 μm, DB-5 (Thermo Fisher Scientific). For detection, an ISQ-QD GC-MS was used, equipped with an EI source. The GC settings were: the inlet temperature was 270°C, the carrier gas was He, and splitless sampling and constant current mode were adopted with the column flow of 1.2 ml/min. The MS settings were: the mode was SIM, the ion source temperature was 300°C, the transmission line temperature was 250°C, the electron bombardment voltage was 70 eV, and the solvent delay was 4 min.

### Glehniae Radix value chains Analysis

Firstly, we identified the production link of GR medicinal materials, and then identified the stakeholders in the production process. Then, stakeholders were matched into different links. Finally, VC analysis charts were made according to different stakeholders in different links. Stakeholders in different stages of GR VCs were analyzed with respect to production or sale modes, financial behavior, and value added (in Yuan/kg). Pesticide residues and heavy metal residues are important factors affecting the safety of medicinal materials. All samples collected from different stakeholders were tested for heavy metals and pesticide residues. In order to better study the traceability of medicinal materials, samples were collected from stakeholders such as independent farmers, wholesalers, retailers and pharmacies. The characters, polysaccharide content and XAT content were determined in order to find suitable indexes for comprehensive evaluation of medicinal materials quality.

### Data Analysis

#### Classification Standard of Glehniae Radix

SPSS and Excel were used for data processing and analysis. A rank sum test, correlation analysis, descriptive statistical analysis, and other methods were used to screen indicators of the GR grade. The correlations between the GR grade and various indicators were analyzed.

#### Clustering Analysis

A geographical indication is a mark that identifies a product originating in the territory of a member state or in a region of that territory, and the quality, reputation, or other definite characteristics of the product shall be determined primarily by its origin. Authentic medicinal materials exhibit all essential characteristics related to geographical indications; that is, authentic medicinal materials are typical products of a determined geographical area (Huang et al., 2007). Therefore, the origin (environmental characteristics) of medicinal materials are closely related to their quality.

In the process of investigation, it was verified that different groups along the chain have different evaluation criteria for GR quality. For example, in the eyes of farmers and wholesalers, the quality of medicinal materials is defined by the difference in their length and thickness; in the eyes of Chinese medicine doctors, by their origin and efficacy; in the eyes of consumers, by the appearance and efficacy. In addition, the GR indicator component is not specified in the ChP (2020). There are no clear standards to measure the quality of medicinal materials. Thus, polysaccharide and XAT were selected as the content determination components of GR from different production areas in this study; and the quality of GR was comprehensively evaluated by combining the appearance and characteristics of the medicinal materials.

The polysaccharide and XAT contents in GR from different production areas were used to study the relationship between the origin and quality of medicinal materials. The *K*-means algorithm is an indirect clustering method based on a measure of similarity between samples. The dataset is divided into different categories by an iterative process so that the criterion function for evaluating the clustering performance can be optimized (Ji and Xiao, 2020). The R-tree indexing algorithm was then used to analyze the spatial index using the *K*-means clustering method.

#### Price Forecasting

Fluctuations in the price of medicinal herbs impact production, sales, and consumption. Therefore, price forecasts are necessary to provide price signals for the cultivation and production of medicinal herbs. The autoregressive integrated moving average (ARIMA) statistical model is widely used for time-series predictions owing to its simplicity and flexibility (Wang and Ma, 2021). The linear model proposed by Box and Jenkins for time-series analysis and prediction (Wang and Ma, 2021) can usually be stabilized by first-order and second-order differences for linear and second-order curves, respectively (Duan, 2021). In this study, local and market prices were collected for Chifeng, Inner Mongolia, and Anguo, Hebei, from 2016 to 2020. The ARIMA model in SPSS (IBM, Armonk, NY, United States ) was used to predict and analyze the market prices of GRs in Inner Mongolia.

## Results and Discussion

### Mapping and Analysis of the Value Chains of Glehniae Radix

GR has a long history of cultivation in China. Owing to its favorable geographical and climatic conditions, Chifeng, Inner Mongolia is the main production region, and therefore, Chifeng GR was selected as our primary research target.

**FIGURE 2:**
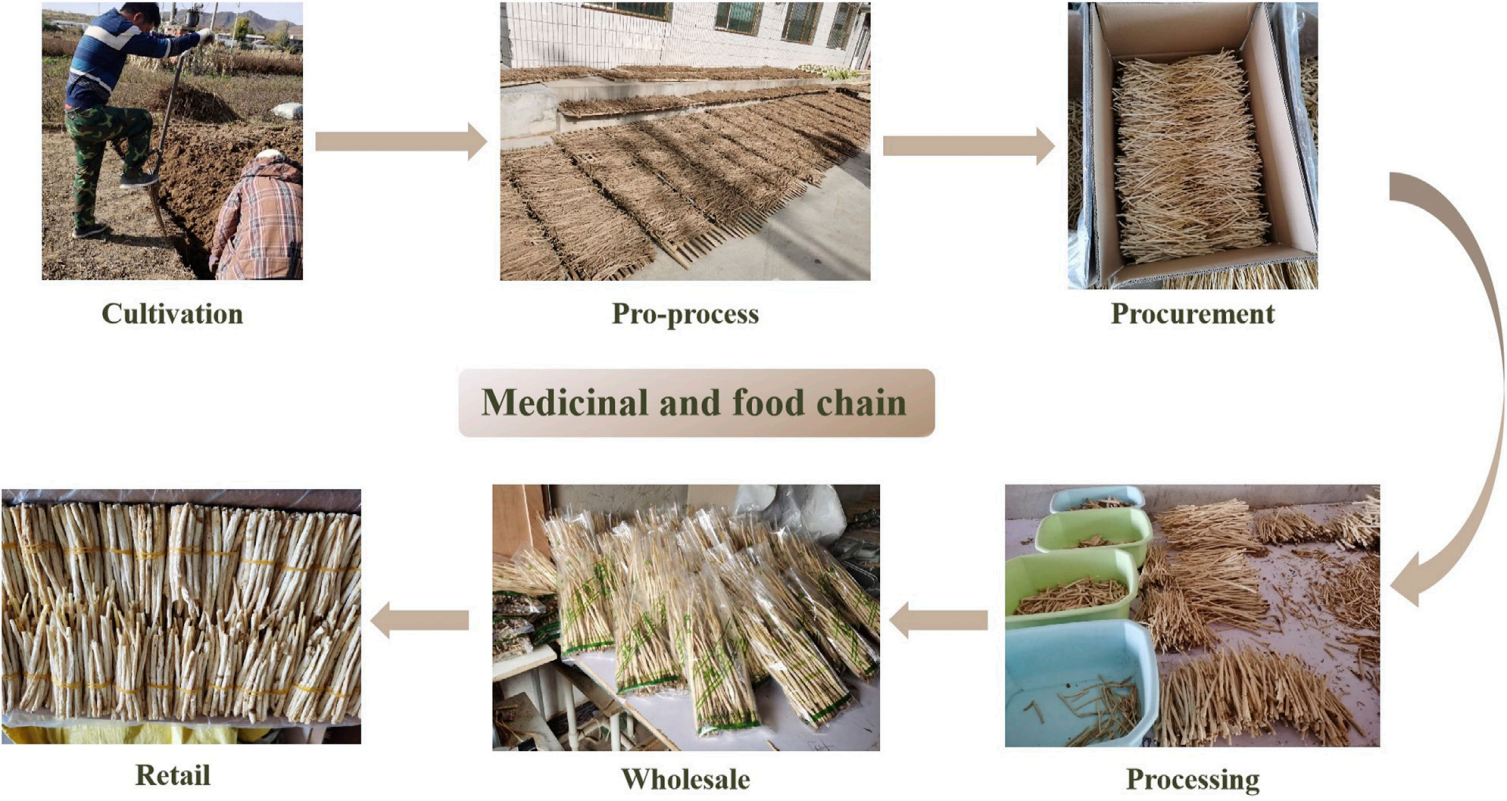
The six stages of GR pathway in the medicinal and food chain.

GR passes through six stages before reaching the final consumer. The first stage is cultivation, which includes seeding, watering, fertilizing, pest management, and harvesting. The second stage is a pro-process, including washing, boiling, peeling, and drying. The third stage is procurement, and wholesalers or herbal piece processing plants make bulk purchases from farmers through brokers. The fourth stage is processing, mainly involving the removal of impurities and slicing according to customer demand—this step makes a distinction between food and medicine; food is often cut into longer segments, and medicine should be cut into small pieces, according to ChP (2020). The fifth step is wholesale, and GR is sold throughout the country through the medicine market. The last step is retail, which includes offline (e.g., food markets, pharmacies, and clinics) and online retail. Different stakeholders with various roles form different VCs. The entire circulation process is illustrated in Figure 2.

**FIGURE 3:**
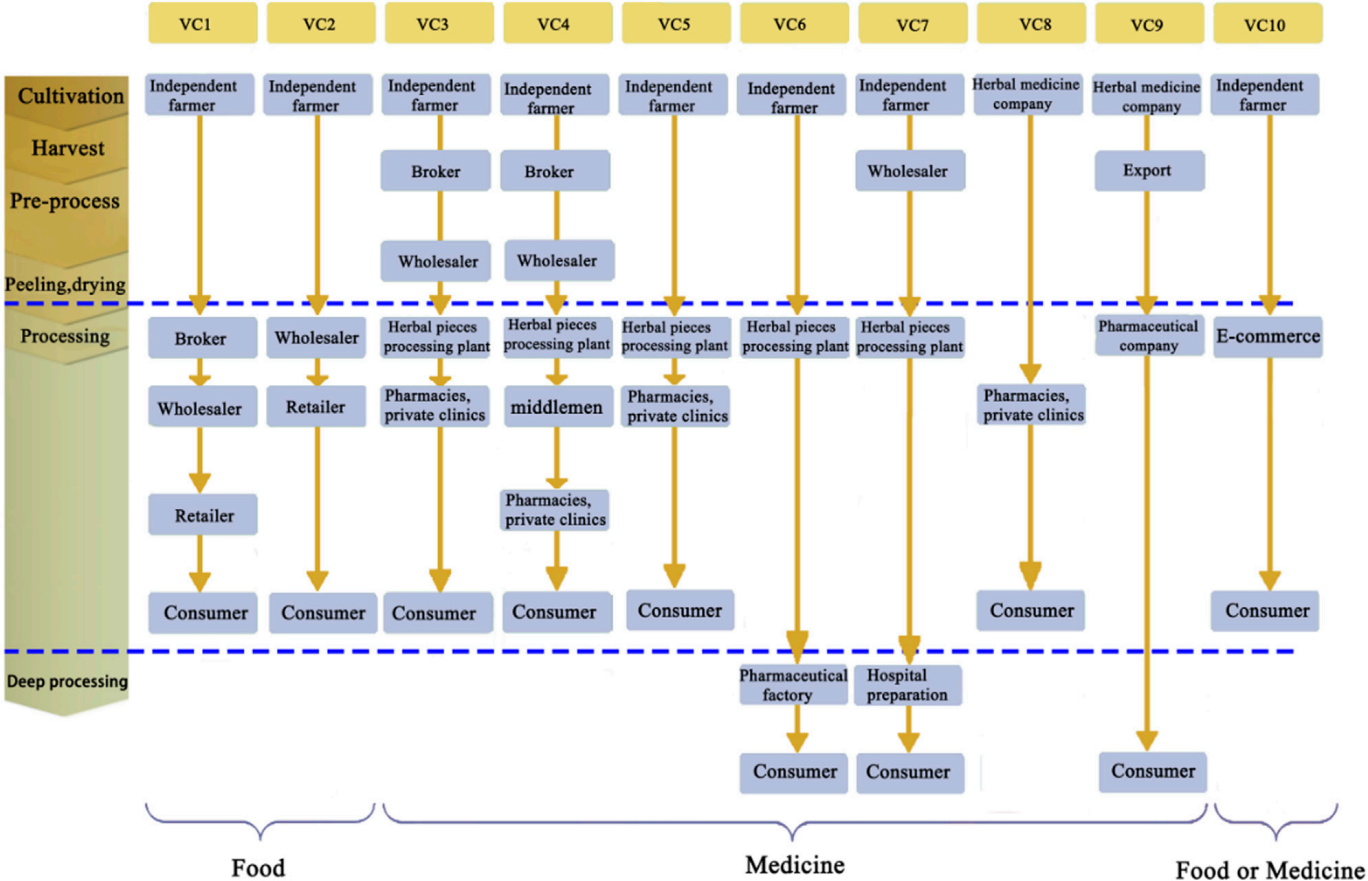
The 10 value chains of GR were drawn after investigation.

A comprehensive VC map with 10 lines indicating different product paths from cultivation to the end consumer was generated, as shown in Figure 3. Most of the VCs are based on independent farmers (VC1–VC7, VC10), and smallholders account for the majority of the GR production. The final products of VC1 and VC2 are food, indicating that processing can be performed by brokers or wholesalers. The final products of VC3–VC9 are medicines (decoction pieces or Chinese patent medicine), and processing or deep processing can only be performed by qualified processing plants or pharmaceutical factories (e.g., GMP certification). VC10 is an emerging circulation channel, and the final product can be both food and medicine; a certain qualification is required to conduct TCM trading.

Independent farmers play a crucial role in the GR planting pattern. In all of the VCs except VC8 and VC9, the processes of cultivation, harvest, washing, peeling, and drying are all handled by independent farmers. VC1 and VC2 are the most primary and general chains, in which farmers sell dried herbs to wholesalers directly or through brokers, and the dried herbs are then transported to TCM markets and circulated around the country. GR used as food is always cut into long segments or sold whole after the removal of impurities, which is performed by wholesalers who utilize brokers or perform the task themselves. Processed GRs are simply packaged or bundled in a small bundle and sold in supermarkets or farmer markets.

VC3–VC5 are typical and traditional medicinal VCs, different from VC1 and VC2, and wholesalers resell GR to herbal processing plants (generally as segments of 0.5–1 cm). After ensuring the quality of the finished products, they are packaged to a certain specification, and brand information is added, which adds value. Processed GRs are sold to hospital pharmacies, retail pharmacies, or private clinics and prescribed to patients by physicians. Medicinal GR has an unattractive appearance compared to that of GR used for food; however, its quality should meet the requirements of ChP (2020). Processing plants handling large herbal pieces also procure GRs directly from local farmers (VC5), increasing ease and flexibility.

TCM preparations are the main form used for therapeutic purposes. Processed GR is also used in herbal medicine preparations to obtain synergistic effects. Pharmaceutical factories or hospitals requiring high quantities of processed GR purchase it from processing plants for herbal pieces, followed by substantial processing for herbal medicine preparations (sold in drugstores) or hospital preparations (only sold in hospitals that produce them). The final products then reach patients through prescribing physicians. VC6–VC7 described the above modes of supply.

In VC8, local TCM companies replace the role of farmers. They rent more land than independent farmers do (by at least 10-fold), hire workers for cultivation, harvesting, and pre-processing, and then process herbs in their own herbal processing plants. Processed GR (decoction pieces) is then sold to hospital pharmacies and private clinics. VC8 is an example of partial vertical integration, and local TCM companies take on the roles of independent farmers, brokers, wholesalers, and processing plants in the above-described VCs, simplifying the VCs. It is a beneficial integration from the perspective of local TCM companies; they can control the quality and supply of GR more directly, rationally allocate resources, and maximize revenue. Employees (mostly local farmers) have a fixed income with little risk involved and can look for other jobs during the slack season. VC8 optimizes the GR supply mode, and TCM companies can implement science-based planting techniques, obtain extensive market information, and are more capable of coping with price fluctuations.

VC9 is an export VC, similar to VC8, in which local TCM companies integrate the process of cultivation, harvest, and pre-process to ensure the quality of GR. The quality requirements for exported GR are higher than those for domestic production. Peeling is not required, and the length and diameter must meet certain requirements. The main import country is Japan; pharmaceutical companies in Japan approach local TCM companies directly, with very few intermediate participants. Exported GRs are highly processed before reaching consumers. As in VC8, local TCM companies can control the use of pesticides by partial vertical integration, thereby ensuring the safety and quality of GR, resulting in higher returns.

The trade of TCM based on the internet (e-commerce) is increasingly common, and the future trend involves a shift in the trade center from the market to the place of origin. VC10 is a typical VC in which e-commerce traders purchase pre-processed GR directly from independent farmers for sale on online platforms, including pharmaceutical factories, processing plants, and retail. E-commerce is currently not a mature supply mode; traders and local farmers have not built a mature cooperation model, the sale channels are not fully established, and sales are far fewer than offline sales are. Moreover, the application practice of this kind of VCs is restricted, and there is no conclusive evidence to show that this kind of transaction mode can guarantee the stability of the quality of GR. Therefore, a further in-depth study is needed to address these issues.

### Characterization of Stakeholders in Glehniae Radix Value Chains

Stakeholders at different stages play distinct roles in creating added value through labor and non-labor inputs. Cultivation and processing are the two main stages adding value in the GR VC; the two stages involve most of the labor input. Others, such as capital input and technical input, are concentrated mainly on the trading stage.

#### Independent Farmers

The growth cycle of GR is 1–2 years. The harvest and processing of GR are time-consuming and laborious. The root of GR is slender with a length of about 20–30 cm, and it easily breaks off when uprooting, requiring substantial physical effort. Although excavating machines have recently become popular, manual operation is still the mainstream harvest method. The most common forms of GR processing include cleaning, boiling in water, peeling, and drying. Peeling is the most complicated and time-consuming step requiring manual operation. After peeling, GR is white, making it more attractive to buyers. No peeling is required for export products. Because it is difficult to achieve mass production, GR farming remains small-scale, primarily involving small independent farms. Most planting areas are about 1–2 ha, with a few farmers planting 4–6 ha. The cultivation often takes the family as a unit and rarely employs workers; therefore, households with more labor force may choose to plant more GR. For most farmers, GR is not the main crop plant; some households with one or two labor forces usually do not plan GR. The inputs and costs consist of field management, harvesting, peeling, and drying. For most farmers, manual harvesting is performed using special tools implemented by the farmers themselves; accordingly, there are no extra costs. Mechanical excavation has emerged in recent years; the rent is ca. 10 Chinese Yuan/kg, and the process is highly efficient (i.e., three times as efficient as manual labor). Peeling is also labor-intensive and requires manual operation. Some farmers hire workers for this task; the cost is two Chinese Yuan/kg, and a worker can process 100 kg per day. Peelers are frequently elderly individuals or women in the village who perform short-term work during the annual harvest season. Other inputs are non-labor costs, including land rent, seeds, fertilizer, and watering. The stages from cultivation to peeling are the most labor-intensive and determine the quality of GR. Most of the added value is created during these stages. However, in the GR VCs, the profits for small GR farmers are relatively low; the farmers are passive in the process of trading owing to the information delay, weak bargaining power, and inability to predict yield and price (Lee et al., 2012).

#### Brokers

Most brokers are native; they mainly serve as a bridge between small farmers and wholesalers and earn intermediate fees. Brokers have stores called distribution stations. They tend to deal with all kinds of local medicinal herbs; however, in most cases, there is no overstock. The brokers organize medicinal materials from farmers according to the needs of customers; only if the market is good, they will choose to store a portion of the cargo. The business scope of brokers mainly includes three aspects: 1) collecting supply and demand information from wholesalers and farmers, acting as a bridge between them. They charge wholesalers intermediate fees of approximately 500–1,500 Chinese Yuan/ton. The higher the free, the more services are provided, such as accommodation, transportation, and the cost of packing; 2) providing GR seeds to farmers at a fee of 20–30 Chinese Yuan/kg. They buy seeds from farmers who have redundant resources and resell them to farmers. Before reselling, they conduct germination tests to ensure the quality of the seeds; 3) providing technical guidance at a fee of 300–500 Chinese Yuan/day. The costs and inputs of the brokers mainly include the cost of transportation, accommodation, and sometimes the package fee. A broker requires reliable, useful information and well-established relationships with the farmer and wholesaler.

#### Wholesalers and Middlemen

The term “wholesaler” refers to an individual engaged in the wholesale of GR, mainly from the large pharmaceutical market, and the throughput is measured in tons. The term “middleman” refers to an individual between wholesalers and retailers, and the scale of operation and working cash is smaller than that of wholesalers. The wholesalers in this study were mainly based at the Anguo or Bozhou medicinal material distribution centers and had fixed stores in the center. They had regular customers, and GR was sold for use in medicine and food products. For culinary use, the dried medicinal materials are processed into sections according to customer demand, or the broker performs processing. The GR used for medicine are sold directly to TCM companies or herbal processing plants for piece processing or deep processing. According to ChP (2020), only enterprises with GMP qualifications can produce herbal pieces. The costs and inputs of wholesalers include rent, traffic expenses, mat endowment, and they pocket the difference.

#### E-Commerce

With the gradual increase in e-commerce, there is a trend toward a shift in the trade of TCMs to the place of origin from the market, achieving a seamless connection between online and offline activity. The operating cost is low and, thus, e-commerce is increasing. E-commerce merchants establish a direct connection to GR farmers or planting firms and sell materials through the network. The new supply mode reduces the number of stages, and retailers or consumers can obtain GR at a lower price. The costs and inputs of e-commerce are mainly the maintenance costs of the platform. This form of commerce is still not mature and requires further investment into channel expansion.

### Link Between Behavior, Product Quality, and Benefit Distribution

The circulation modes of two typical GR VCs are elaborated here to illustrate the effects of different VCs on the quality of GRs and the welfare of stakeholders. Type 1 is an example of high-quality GR, mainly for export, with effective traceability and high returns, corresponding to VC9. Type 2 is the traditional GR VC, which mainly supplies the domestic market for food and medicine. It is the most common processing and selling mode of GR, corresponding to other VCs, except VCs 9 and 10. The links between behavior, product quality, and benefit distribution are outlined below.

Type 1 is the integration of the export of venture enterprises, stricter standards, higher quality and relatively higher returns. VC9 is an excellent example of how each link must strictly control the production process. The complete process for the export of GR, from planting to product packaging, must comply with the standards of the importer. A GR traceability system is also necessary for the standardization of every step in the production process. We conducted a questionnaire interview with university professors in South Korea and Japan to inquire about the export of GR. South Korea imports medicinal materials directly from the country of origin, while Japan buys GR through cooperative import. Online trading occurs in South Korea but not in Japan (and will not be considered in the near future). In both countries, there must be fixed suppliers who are tested according to national pharmacopeia standards for import. In addition, imported GR is mostly used as TCM. Compared with traditional risk investment, investment in this kind of derivative product with high quality and reasonable prices is also greater. A company producing high-quality GR will increase human resources and financial resources to obtain high returns. The reward is not only financial but also includes a favorable corporate reputation and status in society, referred to as intangible value. In South Korea, there is network trade (e.g., VC10); accordingly, quality control in e-commerce should be assured in the future, as e-commerce provides a convenient and fast new method for sales, applicable to any drug import and export.

Type 2 (corresponding to VC1–VC8 in this article) is a traditional GR VC supplying domestic hospitals, clinics, drug stores, or food retailers. In VC1–VC2, GR is sold as food. From the point of view of consumers, they pay more attention to the appearance and characteristics of products and tend to think GR, which is bigger and rounder, is more suitable for soup. Therefore, this kind of medicine is used as food, with better quality and higher price. In VC3–VC7, GR used as medicinal material has stricter quality requirements than GR used as food. The quality of herbs in these VCs is mainly influenced by independent farmers, due to the lack of uniform planting techniques and field management standards, which limits the consistency of GR quality. In terms of safety, the use of chemical fertilizers and pesticides is still unavoidable in the actual planting process. In addition, although the use of DDT has been completely banned in China in 1983, DDT degrades slowly in soil, which will have an impact on medicinal materials (Xiao et al., 2021). Therefore, heavy metals and pesticide residues in the soil became inevitable problems, which affected the quality of GR. As a result of standards and regulations, there are stronger controls on controlled substances and substandard drugs for pesticide abuse. Therefore, independent farmers have a strong sense of management of this aspect in the planting process. So most of the products in different parts of the VCs are safe. Therefore, all 48 samples we collected were tested qualified. Preprocessing of the product is often done by the farmer himself, which is another source of inconsistent GR quality. Due to the uneven quality of medicinal materials and the market-driven environment, the price of medicinal materials mainly depends on the market rather than independent farmers. Meanwhile, due to the lack of unified commodity-grade standards, the price information of independent farmers is updated slowly. Wholesalers hold the bargaining power, and farmers can only choose to sell or not to sell. If market conditions are bad, independent farmers will choose not to harvest, fail to recoup their early investments, or next year’s prices are hard to predict. Independent farmers, on the other hand, unable to establish the traceability system, and the expected income low, small farmers will be looking for ways to reduce the production cost to gain more benefits, these will affect the quality of medicinal materials, making their products only at a low price to enter the low-end market, eventually make their lower profits. Brokers have less impact on the quality of the product, providing only a bridge between supply and demand. Wholesalers and intermediaries will screen the medicinal materials according to their needs. For example, they will select qualified medicinal materials with long length, high polysaccharide content, and heavy metal pesticide residues to process and package them, so that the quality of the medicinal materials can be guaranteed and higher prices can be obtained. In addition, in VC6–VC7, processing plants focus on deep processing to seek a larger market and higher profits. These related plants use a variety of processing methods to develop product added value. In addition, quality products are the key to the development of the factory. To further enhance the product space in the market, the product quality is better and the profit is higher. Moreover, the quality is guaranteed after deep processing. For local Chinese companies in VC8, unlike independent farmers, the quality of Chinese medicinal materials is traceable, and marketing is more diverse, which is reflected in the price of the medicine. Furthermore, brands for this type of medicinal material stabilize and increase the price of medicine. VC8 is an advanced and efficient method to ensure the quality of medicinal materials and the income source of independent farmers and is expected to be the future trend in the processing and selling GR medicinal materials.

### Grade and Quality of GR

#### Grade of Medicinal Materials

**FIGURE 4:**
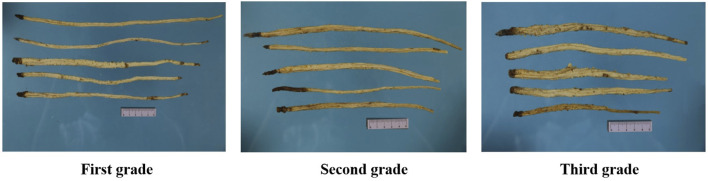
The specification and grade of GR.

Different GR herbs differ in appearance and quality and, therefore, in price. According to the market survey, the grade of GR in the market can be broadly classified based on various indexes, including growth year, upper-middle diameter, and length of the medicinal material. For example, GR that has been growing for 2 years can be divided into large strips (selected goods) exceeding 1 cm in diameter and small strips (unified goods) between 0.6 and 0.8 cm in diameter. However, a unified standard for GR grade classification in the market is lacking. We conducted in-depth research on collected samples of medicinal materials. According to Commodity Specifications and Standards for 76 Kinds of Medicinal Materials and the actual market conditions, the length and the diameter of the upper-middle part of medicinal materials were selected as the appearance-related indexes, and difference analysis of these indexes was performed between the selected goods and the unified goods. There was a significant difference in length but not in the diameter of the upper-middle part. We did not detect a significant correlation between the diameter and length of the upper-middle part; however, there was a significant correlation between the polysaccharide content and the diameter and length of the upper-middle part of medicinal materials. The results showed that the grades of medicinal materials changed from first grade to third grade, the length of medicinal materials decreased gradually, and the content of polysaccharide decreased gradually. Therefore, length was finally selected as a trait index, the upper-middle diameter as an auxiliary index, and the polysaccharide content as an internal index for the classification of GR grade. In addition, xanthotoxin was listed as a carcinogen, so we do not choose this index as the grade index of medicinal materials. As the heavy metals and pesticide residues in medicinal materials in different regions are randomly distributed, so the contents of heavy metals and pesticide residues in different medicinal materials are not regular. Therefore, there is no correlation between the grades and heavy metals and pesticide residues in GR. At the same time, unreasonable pesticides and heavy metals will harm consumers’ health. In this context, it is necessary to ensure that the contents of heavy metals and pesticide residues in GR of different grades are lower than the standards of European pharmacopoeia and ChP to ensure the safety of medicinal materials. The GR was divided into three levels, as shown in Figure 4. See Table 1 for details of the specific classification standards for medicinal materials. It is expected to provide a more scientific and specific classification standard and provide a more reasonable reference basis for the grading standard of GR in the future.

**TABLE 1:** Standard for specification and grade of GR.

Grade	Shape	Surface characteristics	Colour	Length	Upper-middle diameter	Polysaccharide content/%
First grade	Slender and cylindrical	All have fine longitudinal wrinkles and longitudinal furrows, and have brownish yellow dot fine root marks. The apex is usually left with yellowish brown rhizome residues. The strip shape is slender, and the thickness of upper, middle and lower parts is even	Pale yellowish white	≥23 cm	≥0.6 cm	≥13.0%
Second grade		All have fine longitudinal wrinkles and longitudinal furrows, and have brownish yellow dot fine root marks, occasionally residual skin.	Pale yellowish white to yellowish brown	14–23 cm	≥0.6 cm	8.5–13.0%
Third grade		The apex is often left with yellowish - brown rhizome residues. Taper at the top, slightly thick in the middle, tapering at the bottom	Yellowish brown	≤14 cm	≥0.6 cm	≤8.5%

#### Analysis of Quality

Differences in production and processing modes among VCs have different degrees of impact on the quality of medicinal materials. The traceability of the quality of medicinal materials, as an effective and safe quality inspection system, has always been an important aspect of ensuring the quality of medicinal materials (Aung and Chang 2014). However, VCs differ with respect to the traceability of the quality of medicinal materials. Except for VC8 and VC9, the planting and harvesting steps in all other VCs are completed by independent farmers themselves, which can lead to uncertainty in quality. Once sold, it is challenging to ensure traceability. Medicinal materials in VC3–VC7 are processed by factories. During processing, medicinal materials are strictly monitored, and quality traceability is better. In VC10, sources are uncertain, and the traceability of quality cannot be guaranteed. VC8 and VC9 show high traceability and, thereby, reliable quality, as they have a complete and standardized planting, harvesting, and processing system.

**FIGURE 5:**
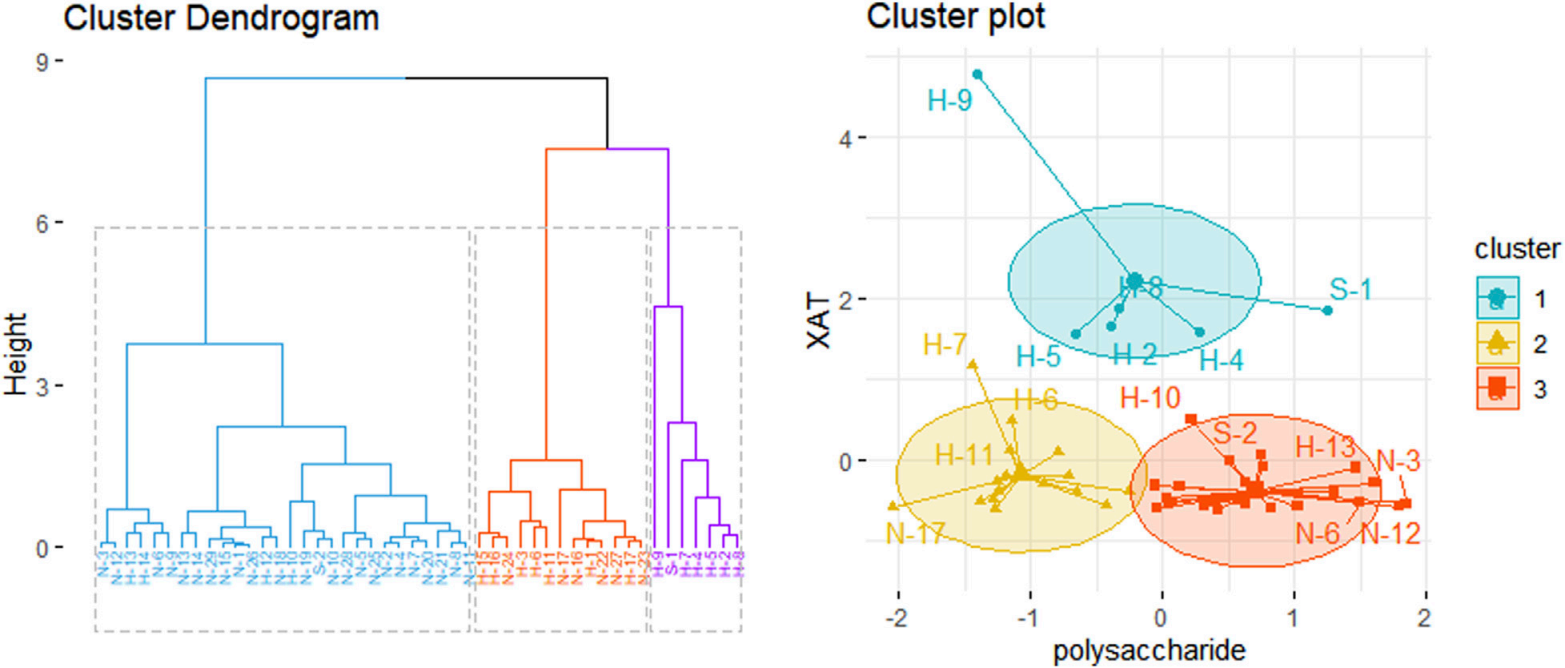
*K*-means clustering results.

Modern analytical techniques can be used to evaluate one aspect of the quality of medicinal materials. We studied medicinal materials from Chifeng, Inner Mongolia, Anguo, Hebei, and Laiyang. Samples were randomly collected from stakeholders in 10 VCs, such as independent farmers, TCM companies, wholesalers, mediators, and retailers, and content determination was performed on the collected samples. The results of various indicators are shown in Supplementary Table S1. Heavy metal and pesticide residues were all qualified. The results of cluster analysis are shown in Figure 5. The cluster results showed that the samples could be divided into three categories. The results of clustering and content determination showed that the polysaccharide content of the three types of samples was very high, and there were differences among different producing areas, among which the content in Inner Mongolia was generally high. So the quality of Inner Mongolia medicine is good. The results of XAT showed that the differences between clustering results were evident, and the relationship between content and region could not be clearly identified.

Content determination and cluster analysis showed that the polysaccharide content in GR was higher than that in XAT. Therefore, the results showed that polysaccharide had the most significant effect on GR quality. Compared with Hebei province, GR in Inner Mongolia had higher polysaccharide content. Meanwhile, GR from Inner Mongolia was better than that from Hebei by measuring the length and upper-middle diameter of medicinal materials. Therefore, the comprehensive evaluation of the quality of GR revealed that the medicinal materials from Inner Mongolia are better than those from Hebei. Further, the price of medicinal materials is often higher in Inner Mongolia than that of other regions, which is consistent with the results of our market survey. According to the comprehensive evaluation of GR indexes (e.g., appearance, content determination), and the quality of Inner Mongolia GR is high. In addition, XAT has been classified as a carcinogen, but there are only animal toxicity studies and few clinical studies. It should be noted that the effects observed in animal models may differ from those in humans, rendering human clinical trials essential. Our study showed the content of XAT in different medicinal materials from different regions. We believe that due to the carcinogenicity of XAT, specific limited studies are needed. Therefore, future research should focus on the limit of XAT content.

### Price Fluctuations and Forecast

**FIGURE 6:**
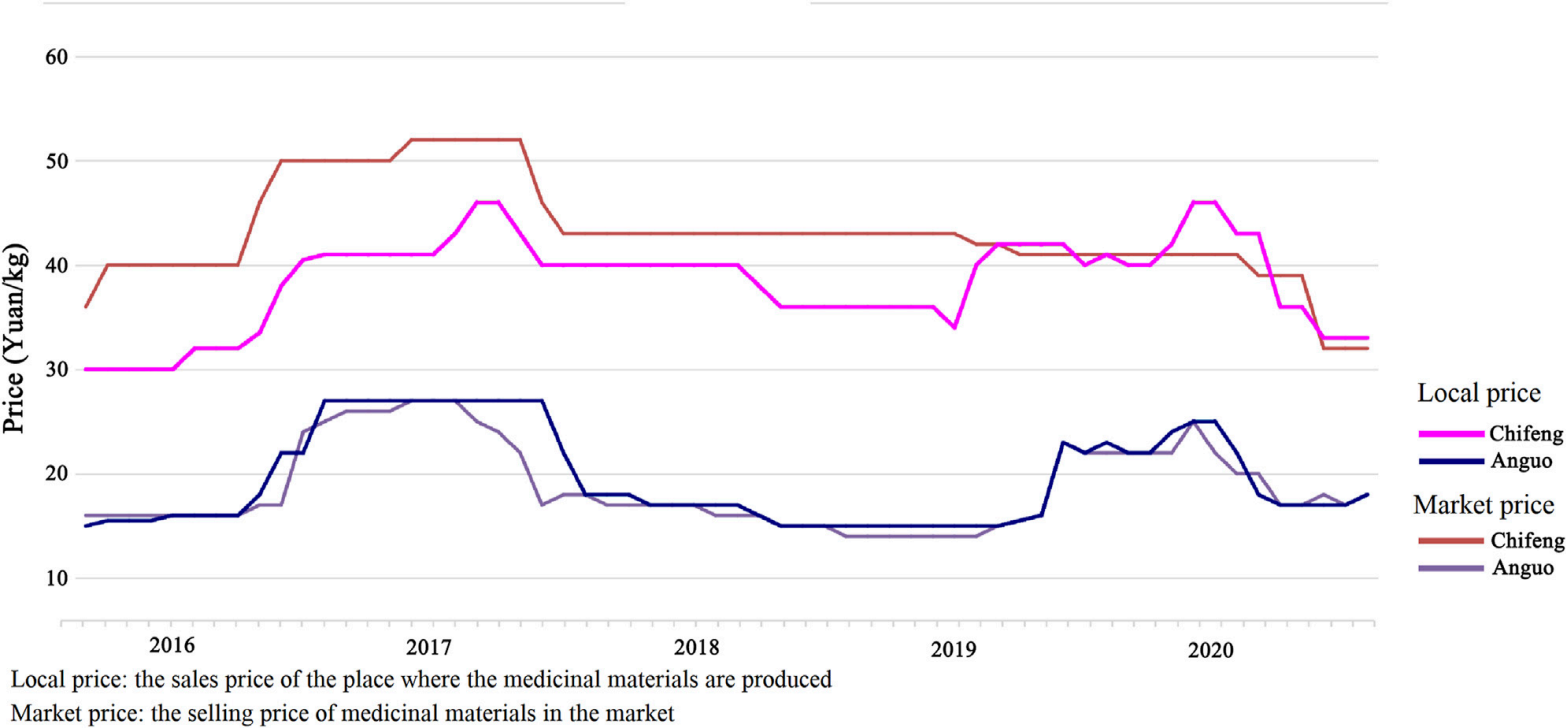
Price details of two regional GR.

In addition to differences in GR quality among production areas, the price also differs. As shown in Figure 6, the price of Inner Mongolia GR is almost double that of Hebei GR. The price of GR from the same production area fluctuates temporally. We collected price data for GR over a period of 5 years and found that the price of unprocessed GR (dried GR) is not fixed from year to year or even month to month. Price fluctuations will affect the stability of the market. From the perspective of farmers, price fluctuations have a significant impact on income. Price uncertainty may lead to suboptimal planting decisions, which will damage profits. For wholesalers, price uncertainty can result in a backlog of goods or a low profit. In addition to describing the trend in price fluctuations, we predict future price trends and provide a reference for relevant stakeholders.

**FIGURE 7:**
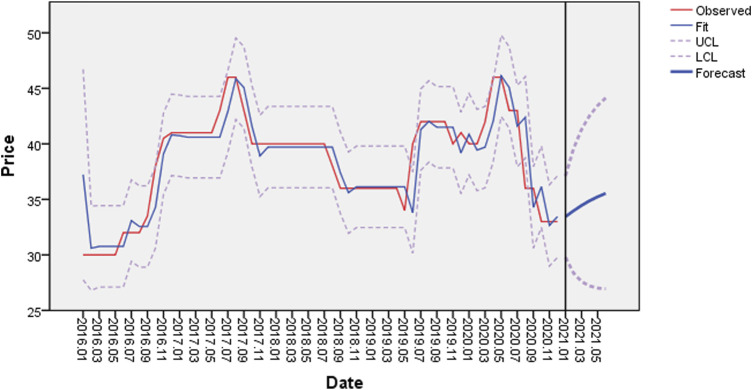
GR price forecast results.

The forecast structure of the market price of GR in Inner Mongolia is shown in Figure 7 (where the horizontal axis represents the date from January 2016, the vertical axis represents the price of medicinal herbs, the blue line shows the forecast trend results for the future price and confidence intervals for price forecasts are indicated by dashed lines, and the price after January 2021 is the predicted price). The price fluctuated substantially in recent years and was generally distributed between 25 and 50 Yuan/kg.

According to the ARIMA price prediction model, the market price of GR in Inner Mongolia is expected to show an upward trend in the future; however, the overall change is not expected to be substantial. This may be explained by the gradual improvements in the production, processing, and sale chain of GR, thereby limiting price fluctuations.

## Conclusion

In this study, the VCs, quality, and prices of GR from different production areas were studied. The results showed that stakeholders in the VCs would have a certain influence on the quality and price of GRs, either promoting or inhibiting the quality and price of GR. Moreover, there were significant differences in the quality and price of medicinal materials from different production areas.

Our analysis of 10 VCs of GR revealed that the entire system could essentially be described as a closed-loop system from planting. Good planting conditions and planting environments are the basis and key to ensuring a high GR quality. Subsequent processing and sales have a partial impact on quality. GRs are sold for different purposes, divided into different grades or for food or medicine, and eventually reach consumers through different channels. Consumers make purchases according to their own needs and consider price comprehensively. Eventually, the benefit gained by the GR, as impacted at the primary production level, indirectly affects the decision of growers to continue producing GR. The importance of growing GR is an essential part of the cycle by protecting the incomes of farmers and the economy. However, it is necessary to ensure the quality of medicinal materials in key processes, such as planting, harvesting, and processing. The quality of medicinal materials is essential for the classification of different grades of medicinal materials. Compared to low-quality medicinal materials, high-quality ones will have better curative effects and be sold at a higher price.

According to this study, there is a common problem in which the benefits of small stakeholders are relatively low compared with their pay. Fluctuations in the market seriously affect the income of small stakeholders. Partial vertical integration is an excellent way to resolve this issue. For example, VC8 and VC9 can achieve a more efficient, standardized, and stable process than those of other VCs through the integration of planting and processing, and some can trace the source of substandard medicinal materials. At the same time, the income of small stakeholders (especially growers) is guaranteed. However, large enterprises will monopolize the market, thereby affecting the livelihoods of scattered sellers who are relatively less influential. This issue should be addressed in the future, and we can learn from the results of partial vertical integration. Cooperation between independent farmers and between farmers and brokers, wholesalers, and retailers can form a rigorous and complete system for planting and purchasing; this ensures that the collected medicinal materials can be sold and that the medicinal materials sold can be traced back to their source.

In this study, statistical analysis was used to classify the specifications of the medicinal materials, hoping to provide a scientific basis for the classification of Chinese medicinal materials in the medicinal material market. The quality of medicinal materials often needs comprehensive evaluation through various indicators. In this study, the results of chemical composition detection and cluster analysis were combined, and the appearance and shape of the medicinal materials were combined to make a comprehensive evaluation of the medicinal materials. The quality of medicinal materials from Inner Mongolia was better than that from the other research areas. The main GR producing area of Inner Mongolia is the unique terrain, climate, farmers’ unique planting and processing technology, which may determine the quality of medicinal materials. In addition, the influence of the environment during cultivation and processing on the quality of the medicinal materials should be explored in more detail in future studies.

Based on collected market prices of Inner Mongolia medicinal materials, the ARMAI model was used for price prediction. The prediction results showed that the price is relatively stable, which may reflect the gradual maturity of the production, processing, and sale chain of GR in recent years and the relatively stable market demand. The price prediction results can be used as a reference for GR growers in the future.

The most crucial aim of this study was to ensure the quality of GR products. VC and stakeholder analyses provided insight into directions for future development and improving the economic benefits for stakeholders. The model of economic development is one of the fundamental driving forces to increase planting; concomitantly, factors affecting the quality of GR should be considered, and the GR for the future of quality control has a certain reference value. In addition, the research on the content limit of carcinogenic substance XAT should also be paid attention to.

## Data Availability

The original contributions presented in the study are included in the article/Supplementary Material, further inquiries can be directed to the corresponding authors.
