# Supplementary material for: Quality Control of Glehniae Radix, the Root of Glehnia Littoralis Fr. Schmidt ex Miq., Along its Value Chains
Source: Front Pharmacol. 2021 Oct 4;12:729554. doi: 10.3389/fphar.2021.729554 (PMC8521048; doi:10.3389/fphar.2021.729554)
Supplement: Supplementary file 1 [file Table1.DOCX]

**Table S1** Details of GR sample

| No. | Origin of medicinal materials | Longitude | Latitude | Length/cm | Upper middle diameter/mm | Moisture/% | Total ash/% | Acid-insoluble ash/% | Extractum/% |
| --- | --- | --- | --- | --- | --- | --- | --- | --- | --- |
| 1 | Changzhuang village, Anguo City, Hebei Province | 115.2786453 | 38.4271071 | 13.71 | 5.22 | 8.95 | 3.86 | 0.55 | 34.97 |
| 2 | \| Dawnv village, dawunv Town, Anguo City \| \| --- \| | 115.2147055 | 38.3782960 | 11.32 | 6.55 | 8.52 | 3.48 | 0.6 | 35.59 |
| 3 | Changzhuang village, Anguo City | 155.2788969 | 38.4269516 | 13.22 | 9.03 | 9.93 | 3.39 | 0.47 | 32.12 |
| 4 | Changzhuang village, Anguo City | 115.2799209 | 38.4282236 | 15.86 | 6.80 | 8.83 | 1.81 | 0.3 | 41.88 |
| 5 | Changzhuang village, Anguo City | 115.2796874 | 38.4275311 | 14.40 | 8.81 | 9.12 | 2.89 | 0.54 | 37.25 |
| 6 | Dongchai village, Anguo City | 115.2455774 | 38.4159213 | 12.40 | 5.29 | 8.22 | 3.33 | 0.49 | 37.11 |
| 7 | Dongchai village, Anguo City | 115.2457211 | 38.4160768 | 13.21 | 5.72 | 8.92 | 3.06 | 0.34 | 30.46 |
| 8 | Dongchai village, Anguo City | 115.2459906 | 38.4159072 | 13.71 | 6.18 | 8.29 | 3.94 | 0.87 | 37.57 |
| 9 | Xiguang village, Anguo City | 115.2690019 | 38.4146741 | 12.41 | 6.67 | 9.41 | 2.96 | 0.48 | 30.64 |
| 10 | Xiguang village, Anguo City | 115.2691636 | 38.4146741 | 13.90 | 7.64 | 8.40 | 4.32 | 0.99 | 48.62 |
| 11 | Xiguang village, Anguo City | 115.2694330 | 38.4150558 | 13.62 | 6.28 | 8.54 | 3.69 | 0.64 | 33.20 |
| 12 | Xiwangqi village, Anguo City | 115.3551897 | 38.3752531 | 21.83 | 8.88 | 8.10 | 2.85 | 0.21 | 53.78 |
| 13 | Xiwangqi village, Anguo City | 115.3551807 | 38.3755077 | 24.39 | 9.09 | 8.64 | 2.78 | 0.15 | 44.87 |
| 14 | Xiwangqi village, Anguo City | 115.3551268 | 38.3753592 | 22.88 | 9.45 | 8.69 | 3.14 | 0.21 | 38.80 |
| 15 | Tantou village, Anguo City | 115.2150219 | 38.3918261 | 14.22 | 7.18 | 8.35 | 4.06 | 0.95 | 28.36 |
| 16 | Tantou village, Anguo City | 115.2155249 | 38.3924059 | 14.31 | 6.46 | 8.79 | 3.54 | 0.66 | 28.52 |
| 17 | Tantou village, Anguo City | 115.2146266 | 38.3917130 | 13.34 | 7.36 | 8.47 | 4.35 | 0.64 | 36.34 |
| 18 | Xiaobo village, Gaogezhuang Town, Laiyang City, Shandong Province | 120.7752241 | 36.7295707 | 18.16 | 6.43 | 8.43 | 2.35 | 0.09 | 45.36 |
| 19 | Xiaoyuan village, Wandi Town, Laiyang City | 120.8833940 | 36.8933389 | 22.93 | 6.39 | 8.53 | 2.18 | 0.06 | 50.46 |
| 20 | Mujianggou, Songshan District, Chifeng City, Inner Mongolia | 118.6544373 | 42.1053128 | 21.77 | 5.88 | 7.96 | 2.17 | 0.13 | 41.05 |
| 21 | Yangshugou village, Songshan District, Chifeng City | 118.2942289 | 42.845563 | 25.31 | 6.30 | 7.94 | 2.5 | 0.11 | 33.42 |
| 22 | Caojiadian hillside, Songshan District, Chifeng City | 118.6414855 | 42.1252959 | 21.73 | 10.78 | 7.77 | 2.42 | 0.14 | 44.79 |
| 23 | Dingjiadi village, Chengzi Township, Songshan District, Chifeng City | 118.7685314 | 42.1866241 | 22.59 | 6.23 | 7.87 | 2.19 | 0.12 | 41.53 |
| 24 | Hillside and flat land of group 4, Shaoguoyingzi village, niuyingzi Town, Kalaqin banner | 118.8252109 | 42.1001561 | 21.51 | 6.74 | 7.74 | 2.22 | 0.18 | 38.4 |
| 25 | Luoyingzi formation 2, niuyingzi village, niuyingzi Town, Kalaqin banner | 118.8013014 | 42.1169371 | 19.81 | 6.65 | 7.53 | 2.17 | 0.07 | 50.2 |
| 26 | North Road natural village, yijiayingzi village, mujiayingzi Town, Songshan District, Chifeng City | 118.8515178 | 42.3437631 | 21.89 | 7.10 | 7.64 | 2.02 | 0.06 | 30.94 |
| 27 | Group 2, shangshuidi village, niujiayingzi Town, Kalaqin banner | 118.8245318 | 42.0867118 | 22.86 | 7.49 | 7.35 | 2.27 | 0.13 | 41.92 |
| 28 | Mayingzi village, Tuchengzi Town, niujiayingzi Town, Kalaqin banner | 118.8478301 | 42.1692573 | 24.37 | 7.39 | 7.86 | 2.02 | 0.06 | 42.07 |
| 29 | Xiaonanyingzi, Shanqian village, niujiayingzi Town, Kalaqin banner | 118.6861084 | 42.1097222 | 24.27 | 6.59 | 7.70 | 2.09 | 0.10 | 35.13 |
| 30 | Group 8, wangyingzi village, niujiayingzi Town, Kalaqin banner | 118.8191016 | 42.1432264 | 19.38 | 6.52 | 7.42 | 2.15 | 0.08 | 41.56 |
| 31 | Zheng yingzi, wangyingzi village, niujiayingzi Town, Kalaqin banner | 118.8177542 | 42.1422637 | 19.40 | 7.62 | 7.46 | 2.61 | 0.22 | 34.29 |
| 32 | Xiaomiaozi village, daniuqun Township, Kalaqin banner | 118.1547459 | 42.3802783 | 19.08 | 6.43 | 7.44 | 2.3 | 0.08 | 25.23 |
| 33 | Yuyingzi village, wangyingzi Town, niujiayingzi Town, Kalaqin banner | 118.8157599 | 42.1424642 | 20.71 | 8.67 | 7.18 | 2.23 | 0.11 | 37.47 |
| 34 | Group 1, Tuchengzi village, niujiayingzi Town, Kalaqin banner | 118.8561378 | 42.1851255 | 22.67 | 6.11 | 7.36 | 2.29 | 0.02 | 22.84 |
| 35 | Yangyingzi, Xishan village, niujiayingzi Town, Kalaqin banner | 118.8534016 | 42.2013889 | 23.26 | 6.63 | 7.16 | 2.92 | 0.07 | 43.32 |
| 36 | Mayingzi village, niujiayingzi Town, Kalaqin banner | 118.8554551 | 42.1850721 | 24.23 | 7.15 | 7.21 | 2.35 | 0.04 | 48.17 |
| 37 | Group 22, daxindi, chenyingzi village, niuyingzi Town, Kalaqin banner | 118.8921123 | 42.2115103 | 26.59 | 5.51 | 7.65 | 2.67 | 0.16 | 18.75 |
| 38 | Xiaoliushu, chenyingzi village, niuyingzi Town, Kalaqin banner | 118.8916991 | 42.2122449 | 23.93 | 6.66 | 7.14 | 2.82 | 0.07 | 49.13 |
| 39 | Wapenyao, chenyingzi village, niuyingzi Town, Kalaqin banner | 118.8916632 | 42.2122315 | 26.11 | 6.71 | 7.72 | 2.16 | 0.02 | 44.23 |
| 40 | Wapenyao, chenyingzi village, niuyingzi Town, Kalaqin banner | 118.8915554 | 42.2123517 | 20.71 | 5.58 | 7.43 | 2.27 | 0.1 | 38.68 |
| 41 | Wapenyao, chenyingzi village, niuyingzi Town, Kalaqin banner | 118.8920854 | 42.2110929 | 20.34 | 5.05 | 7.44 | 2.63 | 0.15 | 39.12 |
| 42 | Group 6, chenyingzi village, niuyingzi Town, Kalaqin banner | 118.8867494 | 42.2112532 | 21.74 | 5.74 | 7.54 | 3.04 | 0.09 | 41.63 |
| 43 | Group 4, chenyingzi village, niuyingzi Town, Kalaqin banner | 118.8875669 | 42.2106387 | 22.21 | 6.37 | 7.67 | 2.75 | 0.08 | 48.99 |
| 44 | Group 6, chenyingzi village, niuyingzi Town, Kalaqin banner | 118.8845306 | 42.2107122 | 27.09 | 7.11 | 7.81 | 2.41 | 0.05 | 42.65 |
| 45 | Goumen, xishuiquan village, Wenzhong Town, Hongshan District, Chifeng City | 118.8835570 | 42.1813598 | 21.48 | 6.53 | 7.68 | 2.46 | 0.1 | 20.76 |
| 46 | Goumen, group 5, xishuiquan village, Wenzhong Town, Hongshan District, Chifeng City | 118.8833414 | 42.1814399 | 21.42 | 5.18 | 7.86 | 2.4 | 0.32 | 45.26 |
| 47 | Group 2, Tiannan village, Tianyi Town, Ningcheng County | 119.3502281 | 41.5999696 | 19.94 | 7.79 | 8.23 | 2.23 | 0.09 | 47.24 |
| 48 | Yushulin village, luolunshu, Tianyi Town, Ningcheng County | 119.3845485 | 41.6088733 | 17.40 | 5.92 | 7.54 | 2.59 | 0.17 | 43.75 |

| No. | Polysaccharide content/% | Xanthotoxin content/% | NO. | Polysaccharide content/% | Xanthotoxin content/% |
| --- | --- | --- | --- | --- | --- |
| 1 | 7.49 | 0.0005461 | 27 | 13.04 | 0.0005078 |
| 2 | 10.14 | 0.0094908 | 28 | 15.2 | 0.0009758 |
| 3 | 8.95 | 0.003008 | 29 | 13.59 | 0.0022936 |
| 4 | 12.14 | 0.0092015 | 30 | 13.21 | 0.0003781 |
| 5 | 9.36 | 0.0090992 | 31 | 16.68 | 0.0001808 |
| 6 | 7.92 | 0.0046254 | 32 | 10.56 | 0.0009387 |
| 7 | 7.02 | 0.0074698 | 33 | 10.06 | 0.0002378 |
| 8 | 10.31 | 0.0104461 | 34 | 11.41 | 0.0006424 |
| 9 | 7.1 | 0.0225999 | 35 | 7.54 | 0.0000883 |
| 10 | 11.98 | 0.0046880 | 36 | 5.21 | 0.0001794 |
| 11 | 7.88 | 0.0031232 | 37 | 11.71 | 0.0012321 |
| 12 | 11.17 | 0.0012958 | 38 | 12.85 | 0.0025286 |
| 13 | 15.66 | 0.0022064 | 39 | 12.28 | 0.0001903 |
| 14 | 16.17 | 0.0014371 | 40 | 13.22 | 0.0014286 |
| 15 | 8.67 | 0.0013909 | 41 | 7.19 | 0.0005097 |
| 16 | 9.21 | 0.0017639 | 42 | 7.58 | 0.0014898 |
| 17 | 7.81 | 0.0017921 | 43 | 9.39 | 0.0010187 |
| 18 | 15.05 | 0.0103114 | 44 | 14.4 | 0.0008202 |
| 19 | 13.55 | 0.0028489 | 45 | 11.55 | 0.0004273 |
| 20 | 11.49 | 0.0003315 | 46 | 7.59 | 0.0010080 |
| 21 | 12.77 | 0.0005903 | 47 | 13.79 | 0.0001054 |
| 22 | 16.83 | 0.0003399 | 48 | 11.19 | 0.0001048 |
| 23 | 12.51 | 0.0004930 |  |  |  |
| 24 | 14.38 | 0.0002017 |  |  |  |
| 25 | 15.79 | 0.0003945 |  |  |  |
| 26 | 12.58 | 0.0000485 |  |  |  |

| No. | [weighing](D:/%E8%BD%AF%E4%BB%B6/Dict/8.9.6.0/resultui/html/index.html#/javascript:;) [sample](D:/%E8%BD%AF%E4%BB%B6/Dict/8.9.6.0/resultui/html/index.html#/javascript:;)/g | The amount measured/ppb (μg/kg) | | | | |
| --- | --- | --- | --- | --- | --- | --- |
|  |  | Cu | As | Cd | Hg | Pb |
| 1 | 0.201 | 0.294 | 0.286 | 0.012 | 0.068 | 0.006 |
| 2 | 0.202 | 2.288 | 0.351 | 0.033 | 0.044 | 0.034 |
| 3 | 0.208 | 3.361 | 0.36 | 0.041 | 0.012 | 0.035 |
| 4 | 0.207 | 3.274 | 0.373 | 0.043 | 0.009 | 0.021 |
| 5 | 0.205 | 2.34 | 0.298 | 0.029 | 0.023 | 0.009 |
| 6 | 0.202 | 2.109 | 0.276 | 0.025 | 0.027 | 0.004 |
| 7 | 0.203 | 1.764 | 0.26 | 0.026 | 0.027 | 0.024 |
| 8 | 0.204 | 1.745 | 0.275 | 0.025 | 0.035 | 0.006 |
| 9 | 0.202 | 1.433 | 0.284 | 0.023 | 0.013 | 0.017 |
| 10 | 0.202 | 1.429 | 0.271 | 0.019 | 0.017 | 0.021 |
| 11 | 0.202 | 1.580 | 0.287 | 0.026 | 0.014 | 0.023 |
| 12 | 0.201 | 1.998 | 0.246 | 0.026 | 0.008 | 0.003 |
| 13 | 0.203 | 2.105 | 0.456 | 0.027 | 0.684 | 0.010 |
| 14 | 0.204 | 2.469 | 0.842 | 0.017 | 2.042 | 0.01 |
| 15 | 0.206 | 2.04 | 0.751 | 0.017 | 1.9 | 0.015 |
| 16 | 0.206 | 1.739 | 0.683 | 0.02 | 1.692 | 0.004 |
| 17 | 0.206 | 1.784 | 0.745 | 0.016 | 1.956 | 0.011 |
| 18 | 0.202 | 0.506 | 0.127 | 0.008 | 0.091 | 0.097 |
| 19 | 0.202 | 0.495 | 0.135 | 0.006 | 0.084 | 0.092 |
| 20 | 0.203 | 0.430 | 0.174 | 0.009 | 0.075 | 0.061 |
| 21 | 0.202 | 3.439 | 0.354 | 0.026 | 0.534 | 0.018 |
| 22 | 0.204 | 10.032 | 0.83 | 0.041 | 1.36 | 0.074 |
| 23 | 0.204 | 10.278 | 0.76 | 0.042 | 1.419 | 0.049 |
| 24 | 0.203 | 9.373 | 0.785 | 0.037 | 1.32 | 0.077 |
| 25 | 0.206 | 8.109 | 0.695 | 0.028 | 1.102 | 0.042 |
| 26 | 0.2 | 8.747 | 0.645 | 0.033 | 1.282 | 0.01 |
| 27 | 0.202 | 8.631 | 0.651 | 0.035 | 1.349 | 0.008 |
| 28 | 0.208 | 7.928 | 0.593 | 0.027 | 1.144 | 0.003 |
| 29 | 0.206 | 7.196 | 0.495 | 0.032 | 0.981 | 0.017 |
| 30 | 0.206 | 7.106 | 0.346 | 0.035 | 0.544 | 0.03 |
| 31 | 0.202 | 6.735 | 0.307 | 0.034 | 0.594 | 0.022 |
| 32 | 0.202 | 7.119 | 0.358 | 0.033 | 0.57 | 0.031 |
| 33 | 0.201 | 6.077 | 0.434 | 0.039 | 0.677 | 0.006 |
| 34 | 0.204 | 6.014 | 0.426 | 0.04 | 0.698 | 0.015 |
| 35 | 0.204 | 5.836 | 0.392 | 0.04 | 0.75 | 0.004 |
| 36 | 0.206 | 7.056 | 0.497 | 0.034 | 1.113 | 0.005 |
| 37 | 0.206 | 6.576 | 0.385 | 0.044 | 0.936 | 0.028 |
| 38 | 0.205 | 5.994 | 0.364 | 0.041 | 0.858 | 0.037 |
| 39 | 0.203 | 5.794 | 0.313 | 0.045 | 0.855 | 0.026 |
| 40 | 0.201 | 5.835 | 0.351 | 0.025 | 0.643 | 0.029 |
| 41 | 0.204 | 5.825 | 0.362 | 0.022 | 0.599 | 0.033 |
| 42 | 0.203 | 5.854 | 0.323 | 0.02 | 0.586 | 0.011 |
| 43 | 0.201 | 2.283 | 0.246 | 0.017 | 0.271 | 0.028 |
| 44 | 0.201 | 2.321 | 0.248 | 0.012 | 0.271 | 0.028 |
| 45 | 0.205 | 4.515 | 0.5 | 0.026 | 0.555 | 0.024 |
| 46 | 0.201 | 4.493 | 0.493 | 0.024 | 0.562 | 0.025 |
| 47 | 0.204 | 4.107 | 0.442 | 0.02 | 0.517 | 0.02 |
| 48 | 0.203 | 3.612 | 0.409 | 0.024 | 0.45 | 0.03 |

| No. | Pesticide residue(mg/kg) | | | | | | | | | | | |
| --- | --- | --- | --- | --- | --- | --- | --- | --- | --- | --- | --- | --- |
|  | Dichlorvos | Trifluralin | *α*-BHC | *β*-BHC | *γ*-BHC | Quintozene | *δ*-BHC | Chlorpyrifos | p,p'-DDE | p,p'-DDD | o,p'-DDT | p,p'-DDT |
| 1 | 0.0033 | 0.0077 | 0.0017 | 0.0073 | - | - | - | 0.0047 | 0.0033 | 0.0048 | 0.0028 | - |
| 2 | 0.0025 | 0.0098 | 0.0005 | 0.0075 | - | - | 0.002 | 0.0062 | 0.0037 | 0.0048 | 0.002 | - |
| 3 | 0.0028 | 0.008 | 0.002 | 0.0073 | - | - | - | 0.005 | 0.005 | 0.0048 | 0.0033 | - |
| 4 | 0.0037 | 0.0097 | 0.0023 | 0.0085 | - | - | - | 0.0047 | - | 0.0058 | 0.0047 | - |
| 5 | 0.0023 | 0.0095 | 0.0043 | 0.0083 | - | - | 0.0156 | 0.0078 | 0.019 | 0.0048 | 0.0032 | - |
| 6 | 0.0037 | 0.0068 | 0.0032 | 0.0065 | - | - | - | 0.0038 | 0.0043 | 0.0043 | 0.0022 |  |
| 7 | 0.0042 | 0.0096 | 0.0052 | 0.0088 | - | - | - | 0.0042 | - | 0.0043 | - | - |
| 8 | 0.0025 | 0.0111 | 0.007 | 0.0123 | - | - | - | 0.0097 | - | 0.0043 | - | - |
| 9 | 0.009 | 0.0117 | 0.0148 | 0.0107 | 0.0017 | - | - | - | - | 0.0053 | 0.0027 | - |
| 10 | 0.0035 | 0.0095 | 0.0093 | 0.0095 | 0.0037 | - | - | 0.1081 | - | 0.0052 | 0.0008 | - |
| 11 | 0.0190 | 0.0080 | 0.0538 | 0.0102 | 0.0233 | 0.0106 | 0.001 | 0.0421 | - | 0.0081 | 0.0054 | 0.0223 |
| 12 | 0.0183 | 0.0081 | 0.0257 | 0.0106 | 0.0345 | 0.0061 | 0.0008 | 0.0131 | - | 0.0043 | 0.0021 | 0.0053 |
| 13 | 0.0184 | 0.0061 | 0.03 | 0.012 | 0.0478 | 0.0031 | - | 0.0114 | - | 0.0042 | 0.0026 | 0.002 |
| 14 | 0.0124 | 0.0062 | 0.0307 | 0.013 | 0.0407 | 0.0014 | - | - | - | 0.0033 | 0.0022 | 0.0166 |
| 15 | 0.0061 | 0.0097 | 0.0405 | 0.0103 | 0.0345 | 0.0024 | - | 0.0235 | - | 0.0081 | 0.0065 | 0.017 |
| 16 | 0.024 | 0.0063 | 0.052 | 0.0167 | 0.0223 | 0.0013 | 0.0013 | 0.003 | - | 0.0063 | 0.003 | - |
| 17 | 0.0157 | 0.0071 | 0.0236 | 0.0177 | 0.0211 | - | 0.015 | 0.0164 | - | 0.0071 | 0.0045 | 0.0034 |
| 18 | 0.0343 | 0.007 | 0.0456 | 0.0166 | 0.0146 | - | 0.001 | 0.0057 | - | 0.0063 | 0.0037 | - |
| 19 | 0.0283 | 0.006 | 0.039 | 0.017 | 0.0163 | - | 0.004 | 0.0053 | - | 0.003 | - | 0.0083 |
| 20 | 0.0336 | 0.0121 | 0.0325 | 0.016 | 0.0203 | - | - | 0.0047 | - | 0.0023 | - | 0.014 |
| 21 | - | 0.0133 | 0.0053 | 0.0137 | 0.0726 | 0.058 | - | 0.0127 | - | 0.0117 | 0.0063 | - |
| 22 | 0.009 | 0.01 | 0.0137 | 0.0256 | 0.028 | 0.0077 | - | 0.006 | 0.0077 | 0.0083 | 0.0063 | - |
| 23 | 0.0097 | 0.0097 | 0.0037 | 0.0047 | 0.0037 | 0.006 | - | 0.006 | 0.0073 | 0.0083 | 0.0063 | - |
| 24 | 0.0093 | 0.009 | 0.0073 | 0.0043 | 0.0136 | 0.007 | - | 0.007 | 0.0073 | 0.0083 | 0.0063 | - |
| 25 | 0.0103 | 0.01 | 0.004 | 0.0243 | 0.0186 | 0.007 | - | 0.006 | 0.0077 | 0.0083 | 0.0063 | - |
| 26 | 0.011 | 0.011 | 0.0027 | 0.0047 | 0.0093 | 0.0063 | - | 0.006 | 0.0073 | 0.0083 | 0.0063 | - |
| 27 | 0.0143 | 0.0093 | 0.002 | 0.0047 | 0.0133 | 0.007 | - | 0.009 | - | 0.0083 | 0.0067 | - |
| 28 | 0.009 | 0.0103 | 0.009 | 0.0223 | - | 0.0063 | - | 0.0063 | 0.0077 | 0.0083 | 0.0063 | - |
| 29 | 0.03 | 0.009 | 0.0107 | 0.0273 | 0.022 | 0.0063 | - | 0.0057 | - | 0.0083 | 0.0063 | - |
| 30 | 0.0087 | 0.0097 | 0.0073 | 0.0353 | 0.014 | 0.006 | - | 0.0063 | 0.0077 | 0.0083 | 0.0063 | - |
| 31 | 0.0146 | 0.0096 | 0.0053 | 0.006 | 0.0083 | 0.0063 | - | 0.0067 | - | 0.0083 | 0.0063 | - |
| 32 | 0.0163 | 0.009 | 0.002 | 0.0053 | 0.0196 | 0.0063 | - | 0.006 | - | 0.0083 | 0.0063 | - |
| 33 | 0.0269 | 0.0086 | 0.0023 | 0.006 | 0.0156 | 0.0063 | - | 0.0067 | - | 0.0093 | 0.007 | - |
| 34 | 0.011 | 0.009 | 0.004 | 0.0047 | 0.0173 | 0.0067 | - | 0.007 | - | 0.0083 | 0.0063 | - |
| 35 | 0.0113 | 0.0093 | - | 0.0053 | 0.0113 | 0.0063 | - | 0.0063 | - | 0.0083 | 0.0063 | - |
| 36 | 0.0087 | 0.0097 | - | 0.0053 | 0.004 | 0.006 | - | 0.0063 | - | 0.01 | 0.0067 | - |
| 37 | 0.0098 | 0.0165 | 0.0028 | 0.0045 | 0.0122 | 0.006 | - | 0.0081 | - | 0.0082 | 0.0063 | - |
| 38 | 0.0106 | 0.018 | 0.009 | 0.0057 | 0.0146 | 0.0067 | - | 0.0077 | - | 0.0083 | 0.0063 | - |
| 39 | 0.0093 | 0.012 | 0.0037 | 0.0047 | 0.0113 | 0.006 | - | 0.008 | 0.0073 | 0.0083 | 0.0063 | - |
| 40 | 0.0186 | 0.0096 | 0.001 | 0.005 | 0.005 | 0.0067 | - | 0.0073 | - | 0.0083 | 0.0067 | - |
| 41 | 0.0207 | 0.0167 | 0.002 | 0.0047 | 0.0067 | 0.0063 | - | 0.0067 | - | 0.0083 | 0.0067 | - |
| 42 | 0.0303 | 0.01 | 0.005 | 0.0047 | 0.0073 | 0.0077 | - | 0.0067 | - | 0.0083 | 0.0067 | - |
| 43 | 0.0096 | 0.0093 | 0.0007 | 0.0043 | 0.0093 | 0.0067 | - | 0.0077 | - | 0.0083 | 0.0063 | - |
| 44 | 0.0093 | 0.0093 | 0.0003 | 0.0047 | 0.0063 | 0.006 | - | 0.006 | - | 0.0083 | 0.0063 | - |
| 45 | 0.01 | 0.0096 | 0.004 | 0.0047 | 0.011 | 0.0063 | - | 0.007 | - | 0.0083 | 0.0063 | - |
| 46 | 0.0065 | 0.0097 | 0.0002 | 0.0047 | 0.0078 | 0.0061 | - | 0.0063 | - | 0.0083 | 0.0063 | - |
| 47 | 0.0103 | 0.009 | - | 0.005 | 0.0017 | 0.0057 | - | 0.0067 | - | 0.0083 | 0.0063 | - |
| 48 | 0.009 | 0.0093 | - | 0.0097 | 0.0103 | 0.011 | - | 0.0063 | - | 0.0087 | 0.007 | - |

- Indicates that the test is not detected
